# Crystal structure of a tetra­kis-substituted pyrazine compound: 2,3,5,6-tetra­kis­(bromo­meth­yl)pyrazine^1^

**DOI:** 10.1107/S1600536814011337

**Published:** 2014-07-19

**Authors:** Tokouré Assoumatine, Helen Stoeckli-Evans

**Affiliations:** a CanAm Bioresearch Inc., 6-1200 Waverley Street, Winnipeg, Manitoba, R3T 6C6, Canada; b Institute of Physics, University of Neuchâtel, rue Emile-Argand 11, CH-2000 Neuchâtel, Switzerland

**Keywords:** crystal structure, tetra­kis-substituted, pyrazine, chiral

## Abstract

The title compound, C_8_H_8_Br_4_N_2_, crystallizes in the enanti­omorphic-defining space group *P*4_1_2_1_2 and has a refined Flack *x* parameter of 0.04 (4). In the asymmetric unit, there are two half-mol­ecules; the whole mol­ecules (*A* and *B*) are generated by twofold rotation symmetry. In mol­ecule *A*, the twofold axis is normal to the pyrazine ring passing through the centre of the ring, while in mol­ecule *B*, the twofold rotation axis lies in the plane of the pyrazine ring bis­ecting the C—C aromatic bonds. The two mol­ecules are pseudo-mirror images of one another, and the best fit of the two mol­ecules was obtained for inverted mol­ecule *B* on mol­ecule *A*, with an r.m.s. deviation of 0.1048 Å and a maximum deviation of any two equivalent atoms of 0.2246 Å. In the crystal, the *A* mol­ecules are linked by weak C—H⋯Br hydrogen bonds and Br⋯Br inter­actions [3.524 (3) Å], forming a three-dimensional framework. The *B* mol­ecules are also linked by weak C—H⋯Br hydrogen bonds and Br⋯Br inter­actions [3.548 (3) Å], forming a three-dimensional network that inter­penetrates the network of *A* mol­ecules.

## Abstract 2

The title molecule crystallizes with two half-molecules in the asymmetric unit, the whole molecules being generated by twofold rotation symmetry. In the crystal, there are two interpenetrating three-dimensional networks involving the individual molecules that are linked by C—H⋯Br and Br⋯Br interactions.

## Chemical context   

The title compound is the starting material used for the synthesis of a number of 2,3,5,6-tetra­kis-substituted pyrazine compounds (Ferigo *et al.*, 1994 ▶; Assoumatine, 1999 ▶). For example, 2,3,5,6-tetra­kis­(amino­meth­yl)pyrazine has been used as a ligand to prepare copper(II), zinc(II) and manganese(II) binuclear and polymeric complexes (Ferigo *et al.*, 1994 ▶).
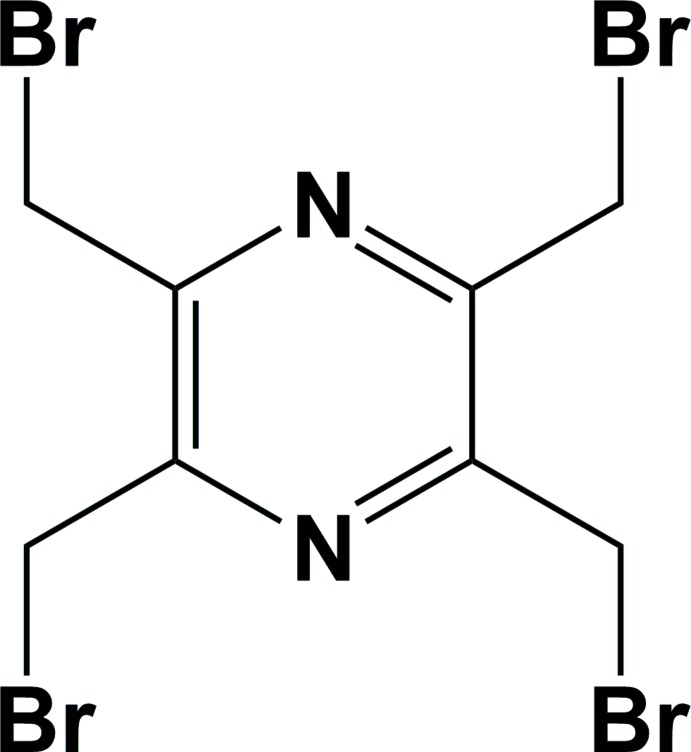


## Structural commentary   

**Figure 1:**
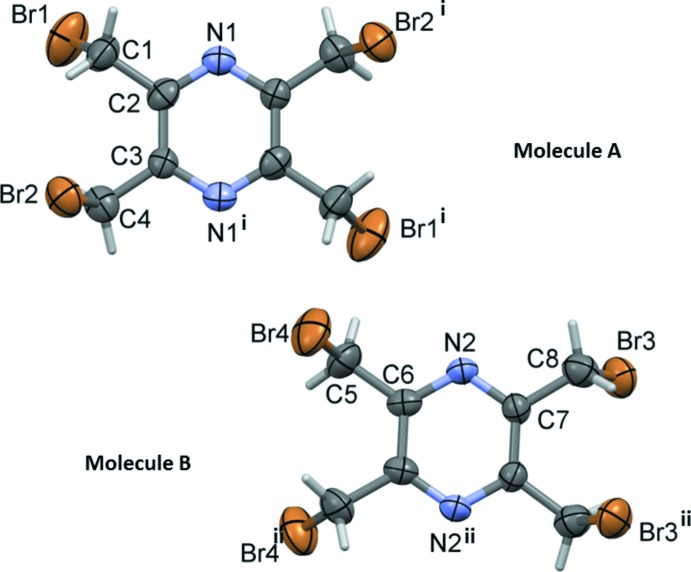
A view of the mol­ecular structure of the two independent mol­ecules (*A* and *B*) of the title compound, with atom labelling [symmetry codes: (i) −*y* + 1, −*x* + 1, −*z* + 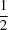; (ii) *y*, *x*, −*z*]. The displacement ellipsoids are drawn at the 50% probability level.

The title compound, Fig. 1 ▶, crystallizes with two half-mol­ecules per asymmetric unit. The whole mol­ecules (*A* and *B*) are generated by twofold rotation symmetry. In mol­ecule *A*, the twofold axis is normal to the pyrazine ring passing through the centre of the ring. In mol­ecule *B*, the twofold rotation axis lies in the plane of the pyrazine ring bis­ecting the C6—6^ii^ and C7—C7^ii^ bonds [symmetry code: (ii) *y*, *x*, −*z*]. Placed side by side, it can be seen that the two mol­ecules are almost perfect mirror images of each other (Fig. 1 ▶). The best fit of the two mol­ecules, calculated using the Mol­ecular Overlay routine in *Mercury* (Macrae *et al.*, 2008 ▶), was obtained for inverted mol­ecule *B* on mol­ecule *A* with an r.m.s. deviation of 0.1048 Å and a maximum deviation of any two equivalent atoms of 0.2246 Å.

The main difference appears for the torsion angles Br1—C1—C2—C3 = −92.6 (15) ° in mol­ecule *A* and Br4—C5—C6—C6^ii^ = 84.8 (17) ° in mol­ecule *B* [Table 1 ▶; symmetry code: (ii) *y*, *x*, −*z*]. The other torsion angles involving the Br—C—C_ar_—C_ar_ (ar = aromatic) arms do not differ significantly (Table 2 ▶).

## Supra­molecular features   

**Figure 3:**
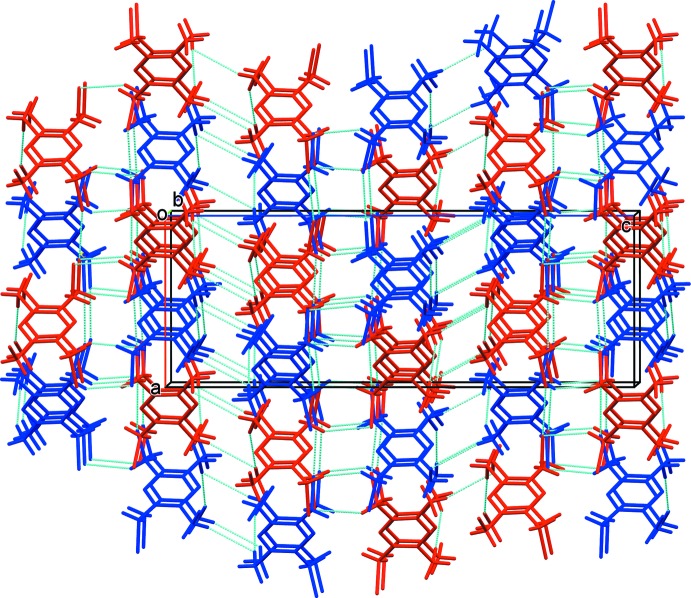
A view along the *b* axis of the crystal packing of the title compound. The C—H⋯Br hydrogen bonds and Br⋯Br inter­actions are shown as dashed lines (see Table 2 ▶ for details; *A* mol­ecules blue, *B* mol­ecules red).

In the crystal, there are two inter­penetrating three-dimensional networks composed of a network of *A* mol­ecules, linked by weak C—H⋯Br hydrogen bonds and Br1⋯Br2^iii^ inter­actions [3.524 (3) Å; symmetry code: (iii) −*y* + 2, −*x* + 1, −*z* + 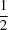], and a network of *B* mol­ecules, are also linked by weak C-H⋯Br hydrogen bonds and Br3⋯Br4^iv^ inter­actions [3.548 (3) Å, symmetry code: (iv) *x*, *y* − 1, *z*] (Table 2 ▶ and Fig. 3 ▶).

## Database survey   

A search of the Cambridge Structural Database (Version 5.33, last update November 2013; Allen, 2002 ▶) indicated the presence of a large number of tetra­substituted pyrazine deriv­atives and their metal complexes, mainly involving tetra­methyl­pyrazine. A small number of them involve 2,3,5,6-tetra­kis­(amino­meth­yl)pyrazine (tampyz), which was used to prepare transition metal binuclear complexes, for example [Cl_2_Zn(tampyz)ZnCl_2_], and a quasi-linear one-dimensional coordination polymer, {Mn(tampyz)Cl_2_·2H_2_O}_*n*_ (Ferigo *et al.*, 1994 ▶). The title compound has also been used in the synthesis of two triclinic polymorphs of 2,3,5,6 tetra­kis­(naphthalen-2-ylsulfanylmeth­yl)pyrazine (Pacifico & Stoeckli-Evans, 2004 ▶), 2,3,5,6-tetra­kis­((naphthalen-2-yl­oxy)meth­yl)pyrazine (Gasser & Stoeckli-Evans, 2007 ▶), 2,3,5,6-tetra­kis­(phen­oxy­meth­yl)pyrazine and 2,3,5,6-tetra­kis­(phenyl­sulfanylmeth­yl)pyrazine (Assoumatine *et al.*, 2007 ▶). All five structures possess inversion symmetry. The sulfanyl derivatives crystallize in the triclinic space group *P* 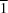, while the oxy derivatives crystallize in the monoclinic space group *P*2_1_/*c*.

## Synthesis and crystallization   

The title compound was prepared by a modification of the procedure described by Ferigo *et al.* (1994 ▶). To 2,3,5,6-tetra­methyl­pyrazine (28 g, 0.28 mol) in CCl_4_ (1 l) was added well-ground *N*-bromo­succinimide (150 g, 0.84 mol). The mixture was stirred vigorously and heated to reflux. As soon as the reflux set in, the mixture was irradiated for 5 h with two 200 W lamps fitted at least 10 cm at opposite sides of the flask. After the mixture was then cooled firstly to room temperature and the floating succinimide filtered off. The orange filtrate was cooled overnight to 278 K to crystallize the remaining traces of succinimide, which was filtered off. The filtrate was evaporated and the residual orange oil dissolved in 50 ml of diethyl ether. This solution was maintained at 278 K for at least one week, whereupon a white crystalline material deposited. The solid was filtered off, then recrystallized in ethanol to give colourless rod-like crystals of the title compound: Yield 7.87 g (8%); m.p. 401–405 K; *R*_F_ 0.54 (toluene/light petroleum, 10/1 *v*/*v*). Analysis for C_8_H_8_Br_4_N_2_ (*M_r_* = 451.78 g/mol); Calculated (%): C 21.27; H 1.79; N 6.20. Found (%): C 21.41; H 1.72; N 6.10. Spectroscopic data: ^1^H-RMN (CDCl_3_, 400 MHz): δ = 4.69 (*s*, 8H, Pz-CH_2_-S) p.p.m.; ^13^C-RMN (CDCl_3_, 100 MHz): δ = 150.41, 28.75 p.p.m. MS (EI, 70 eV), *m*/*z* (%): 452 ([*M* ^+^], 11.9), 371 (100), 292 (13.2), 211 (20.7), 131 (32.7), 92 (20.4), 65 (18.8); IR (KBr disc, cm^−1^): 3030 *w*, 2977 *w*, 1438 *s*, 1405 *s*, 1220 *s*, 1096 *m*, 923 *w*, 787 *s*, 731 *m*, 629 *m*, 596 *w*, 543 *m*, 445 *m*.

## Refinement   

Crystal data, data collection and structure refinement details are summarized in Table 3 ▶. The C-bound H atoms were included in calculated positions and treated as riding atoms: C—H = 0.97 Å with *U*_iso_(H) = 1.2*U*_eq_(C).

## Supplementary Material

Crystal structure: contains datablock(s) I. DOI: 10.1107/S1600536814011337/hb0005sup1.cif

Structure factors: contains datablock(s) I. DOI: 10.1107/S1600536814011337/hb0005Isup2.hkl

Supporting information file. DOI: 10.1107/S1600536814011337/hb0005Isup3.cml

CCDC reference: 1004263

Additional supporting information:  crystallographic information; 3D view; checkCIF report

## Figures and Tables

**Figure 2:**
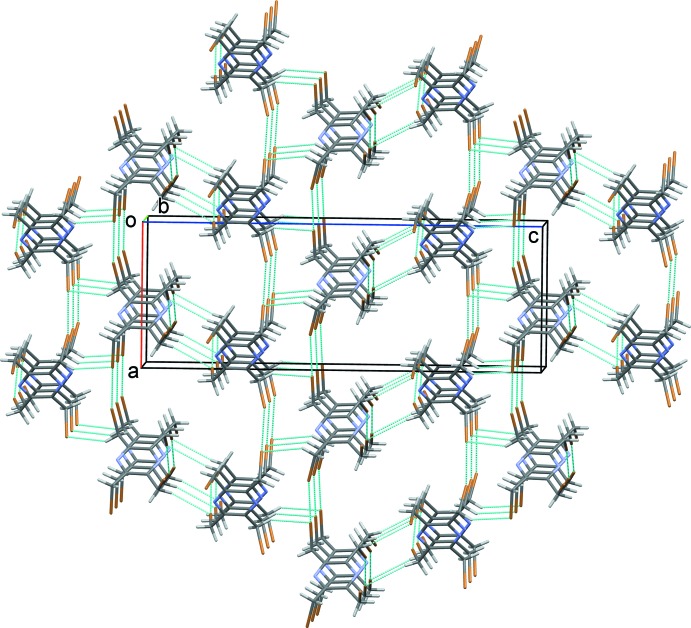
A view along the *b* axis of the crystal packing of the *A* mol­ecules of the title compound. The weak C—H⋯Br hydrogen bonds and Br⋯Br inter­actions are shown as dashed lines (see Table 2 ▶ for details).

**Table 1:** Selected torsion angles (°)

Br1—C1—C2—N1	91.3 (13)	Br4—C5—C6—N2	−93.3 (12)
Br1—C1—C2—C3	−92.6 (15)	Br4—C5—C6—C6^ii^	84.8 (17)
N1^i^—C3—C4—Br2	103.1 (11)	N2—C7—C8—Br3	−101.0 (12)
C2—C3—C4—Br2	−78.6 (15)	C7^ii^—C7—C8—Br3	77.4 (18)

Symmetry codes: (i) 
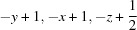
; (ii) 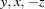.

**Table 2:** Hydrogen-bond geometry (Å, °)

*D*—H⋯*A*	*D*—H	H⋯*A*	*D*⋯*A*	*D*—H⋯*A*
C1—H1*A*⋯Br2^iii^	0.97	3.02	3.863 (14)	146
C1—H1*B*⋯Br2	0.97	2.86	3.617 (16)	135
C5—H5*A*⋯Br4^ii^	0.97	3.04	3.748 (16)	131
C5—H5*B*⋯Br3^iv^	0.97	3.03	3.864 (14)	145
C8—H8*A*⋯Br3^ii^	0.97	2.96	3.654 (15)	130

Symmetry codes: (ii) 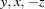; (iii) 
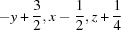
; (iv) 
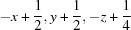
.

**Table 3:** Experimental details

Crystal data	
Chemical formula	C_8_H_8_Br_4_N_2_
*M*_r_	451.80
Crystal system, space group	Tetragonal, *P*4_1_2_1_2
Temperature (K)	293
*a*, *c* (Å)	9.6858 (4), 26.5116 (17)
*V* (Å^3^)	2487.2 (3)
*Z*	8
Radiation type	Mo *K*α
μ (mm^−1^)	12.91
Crystal size (mm)	0.50 × 0.40 × 0.30

Data collection	
Diffractometer	Stoe *IPDS* 1
Absorption correction	Multi-scan (*MULscanABS* in *PLATON*; Spek, 2009 ▶)
*T*_min_, *T*_max_	0.430, 1.000
No. of measured, independent and observed [*I* > 2σ(*I*)] reflections	19463, 2417, 1276
*R*_int_	0.113
(sin θ/λ)_max_ (Å^−1^)	0.616

Refinement	
*R*[*F* ^2^ > 2σ(*F* ^2^)], *wR*(*F* ^2^), *S*	0.043, 0.096, 0.84
No. of reflections	2417
No. of parameters	127
H-atom treatment	H-atom parameters constrained
Δρ_max_, Δρ_min_ (e Å^−3^)	0.69, −0.54
Absolute structure	Flack *x* determined using 419 quotients [(I^+^)−I^−^)]/[(I^+^)+(I^−^)] (Parsons & Flack, 2004 ▶)
Absolute structure parameter	0.04 (4)

Computer programs: *EXPOSE*, *CELL* and *INTEGRATE* in *IPDS*-I (Stoe & Cie, 2004 ▶), *SHELXS97* and *SHELXL2013* (Sheldrick, 2008 ▶), *Mercury* (Macrae *et al.*, 2008 ▶), *PLATON* (Spek, 2009 ▶) and *publCIF* (Westrip, 2010 ▶).

## supplementary crystallographic information

### Crystal data

**Table:**

C_8_H_8_Br_4_N_2_	*D*_x_ = 2.413 Mg m^−^^3^
*M**_r_* = 451.80	Mo *K*α radiation, λ = 0.71073 Å
Tetragonal, *P*4_1_2_1_2	Cell parameters from 5000 reflections
Hall symbol: P 4abw 2nw	θ = 2.2–26.0°
*a* = 9.6858 (4) Å	µ = 12.91 mm^−^^1^
*c* = 26.5116 (17) Å	*T* = 293 K
*V* = 2487.2 (3) Å^3^	Rod, colourless
*Z* = 8	0.50 × 0.40 × 0.30 mm
*F*(000) = 1680	

### Data collection

**Table:**

Stoe IPDS 1 diffractometer	2417 independent reflections
Radiation source: fine-focus sealed tube	1276 reflections with *I* > 2σ(*I*)
Plane graphite monochromator	*R*_int_ = 0.113
φ rotation scans	θ_max_ = 26.0°, θ_min_ = 2.2°
Absorption correction: multi-scan (*MULscanABS* in *PLATON*; Spek, 2009)	*h* = −11→11
*T*_min_ = 0.430, *T*_max_ = 1.000	*k* = −11→11
19463 measured reflections	*l* = −32→32

### Refinement

**Table:**

Refinement on *F*^2^	Secondary atom site location: difference Fourier map
Least-squares matrix: full	Hydrogen site location: inferred from neighbouring sites
*R*[*F*^2^ > 2σ(*F*^2^)] = 0.043	H-atom parameters constrained
*wR*(*F*^2^) = 0.096	*w* = 1/[σ^2^(*F*_o_^2^) + (0.0401*P*)^2^] where *P* = (*F*_o_^2^ + 2*F*_c_^2^)/3
*S* = 0.84	(Δ/σ)_max_ < 0.001
2417 reflections	Δρ_max_ = 0.69 e Å^−^^3^
127 parameters	Δρ_min_ = −0.54 e Å^−^^3^
0 restraints	Absolute structure: Flack *x* determined using 419 quotients [(I^+^)-(I^-^)]/[(I^+^)+(I^-^)] (Parsons & Flack, 2004)
Primary atom site location: structure-invariant direct methods	Absolute structure parameter: 0.04 (4)

### Special details

**Table:**

Geometry. All e.s.d.'s (except the e.s.d. in the dihedral angle between two l.s. planes) are estimated using the full covariance matrix. The cell e.s.d.'s are taken into account individually in the estimation of e.s.d.'s in distances, angles and torsion angles; correlations between e.s.d.'s in cell parameters are only used when they are defined by crystal symmetry. An approximate (isotropic) treatment of cell e.s.d.'s is used for estimating e.s.d.'s involving l.s. planes.

### Fractional atomic coordinates and isotropic or equivalent isotropic displacement parameters (Å^2^)

**Table:**

	*x*	*y*	*z*	*U*_iso_*/*U*_eq_	
Br1	1.2259 (2)	0.2336 (2)	0.31902 (9)	0.0928 (7)	
Br2	0.97546 (18)	0.47643 (17)	0.18421 (6)	0.0654 (5)	
N1	0.8843 (11)	0.1271 (10)	0.3022 (5)	0.043 (3)	
C1	1.0492 (15)	0.3062 (16)	0.3026 (6)	0.058 (4)	
H1A	1.0016	0.3313	0.3335	0.069*	
H1B	1.0608	0.3893	0.2827	0.069*	
C2	0.9639 (14)	0.2070 (12)	0.2742 (5)	0.045 (3)	
C3	0.9590 (13)	0.2017 (12)	0.2214 (4)	0.038 (3)	
C4	1.0501 (16)	0.2865 (14)	0.1876 (5)	0.053 (4)	
H4A	1.0525	0.2463	0.1541	0.063*	
H4B	1.1435	0.2883	0.2008	0.063*	
Br3	0.02090 (19)	−0.23131 (16)	0.06101 (6)	0.0663 (5)	
Br4	0.2379 (2)	0.4746 (2)	0.07000 (7)	0.0854 (6)	
N2	0.1293 (10)	0.1274 (11)	0.0529 (5)	0.042 (4)	
C5	0.3130 (15)	0.2938 (15)	0.0568 (6)	0.060 (4)	
H5A	0.3995	0.3030	0.0387	0.072*	
H5B	0.3317	0.2476	0.0885	0.072*	
C6	0.2145 (13)	0.2082 (13)	0.0262 (4)	0.041 (3)	
C7	0.0442 (13)	0.0470 (13)	0.0261 (4)	0.039 (3)	
C8	−0.0509 (16)	−0.0420 (17)	0.0578 (5)	0.056 (4)	
H8A	−0.1426	−0.0425	0.0432	0.068*	
H8B	−0.0575	−0.0041	0.0916	0.068*	

### Atomic displacement parameters (Å^2^)

**Table:**

	*U*^11^	*U*^22^	*U*^33^	*U*^12^	*U*^13^	*U*^23^
Br1	0.0587 (12)	0.0884 (15)	0.1313 (15)	0.0040 (9)	−0.0371 (11)	−0.0122 (14)
Br2	0.0729 (11)	0.0512 (9)	0.0719 (10)	0.0017 (7)	0.0043 (10)	0.0119 (9)
N1	0.046 (9)	0.046 (9)	0.037 (7)	0.002 (5)	−0.002 (5)	0.002 (5)
C1	0.056 (9)	0.059 (9)	0.058 (9)	−0.009 (8)	−0.009 (8)	−0.011 (8)
C2	0.051 (8)	0.035 (7)	0.050 (7)	0.004 (6)	−0.003 (7)	−0.008 (6)
C3	0.034 (7)	0.037 (7)	0.043 (7)	0.003 (6)	0.001 (6)	−0.003 (6)
C4	0.053 (9)	0.047 (8)	0.059 (8)	0.004 (6)	0.007 (8)	0.002 (7)
Br3	0.0813 (12)	0.0468 (9)	0.0710 (9)	−0.0060 (8)	−0.0011 (10)	0.0101 (8)
Br4	0.0862 (13)	0.0616 (11)	0.1086 (15)	−0.0065 (9)	0.0033 (12)	−0.0330 (11)
N2	0.046 (9)	0.050 (9)	0.030 (6)	−0.006 (5)	−0.007 (5)	0.003 (5)
C5	0.053 (9)	0.073 (10)	0.054 (9)	−0.009 (8)	−0.015 (8)	−0.006 (8)
C6	0.047 (8)	0.043 (7)	0.033 (6)	0.007 (6)	−0.002 (6)	−0.002 (6)
C7	0.036 (7)	0.040 (7)	0.042 (6)	−0.003 (6)	0.000 (6)	0.005 (6)
C8	0.060 (9)	0.062 (9)	0.047 (7)	0.001 (8)	0.000 (8)	0.007 (8)

### Geometric parameters (Å, º)

**Table:**

Br1—C1	1.901 (15)	Br3—C8	1.963 (17)
Br2—C4	1.978 (14)	Br4—C5	1.928 (15)
N1—C2	1.319 (17)	N2—C7	1.337 (15)
N1—C3^i^	1.334 (15)	N2—C6	1.339 (16)
C1—C2	1.474 (18)	C5—C6	1.502 (17)
C1—H1A	0.9700	C5—H5A	0.9700
C1—H1B	0.9700	C5—H5B	0.9700
C2—C3	1.402 (15)	C6—C6^ii^	1.39 (2)
C3—N1^i^	1.334 (15)	C7—C7^ii^	1.39 (2)
C3—C4	1.504 (17)	C7—C8	1.516 (17)
C4—H4A	0.9700	C8—H8A	0.9700
C4—H4B	0.9700	C8—H8B	0.9700
			
C2—N1—C3^i^	117.9 (13)	C7—N2—C6	116.1 (12)
C2—C1—Br1	112.4 (10)	C6—C5—Br4	111.1 (9)
C2—C1—H1A	109.1	C6—C5—H5A	109.4
Br1—C1—H1A	109.1	Br4—C5—H5A	109.4
C2—C1—H1B	109.1	C6—C5—H5B	109.4
Br1—C1—H1B	109.1	Br4—C5—H5B	109.4
H1A—C1—H1B	107.9	H5A—C5—H5B	108.0
N1—C2—C3	121.3 (11)	N2—C6—C6^ii^	121.7 (7)
N1—C2—C1	115.0 (12)	N2—C6—C5	115.4 (11)
C3—C2—C1	123.6 (12)	C6^ii^—C6—C5	122.9 (8)
N1^i^—C3—C2	120.8 (11)	N2—C7—C7^ii^	121.9 (7)
N1^i^—C3—C4	115.3 (12)	N2—C7—C8	114.3 (11)
C2—C3—C4	123.8 (11)	C7^ii^—C7—C8	123.7 (7)
C3—C4—Br2	108.7 (9)	C7—C8—Br3	109.9 (10)
C3—C4—H4A	109.9	C7—C8—H8A	109.7
Br2—C4—H4A	109.9	Br3—C8—H8A	109.7
C3—C4—H4B	109.9	C7—C8—H8B	109.7
Br2—C4—H4B	109.9	Br3—C8—H8B	109.7
H4A—C4—H4B	108.3	H8A—C8—H8B	108.2
			
C3^i^—N1—C2—C3	−0.2 (16)	C2—C3—C4—Br2	−78.6 (15)
C3^i^—N1—C2—C1	175.9 (12)	C7—N2—C6—C6^ii^	4 (2)
Br1—C1—C2—N1	91.3 (13)	C7—N2—C6—C5	−177.8 (12)
Br1—C1—C2—C3	−92.6 (15)	Br4—C5—C6—N2	−93.3 (12)
N1—C2—C3—N1^i^	0.3 (19)	Br4—C5—C6—C6^ii^	84.8 (17)
C1—C2—C3—N1^i^	−175.5 (12)	C6—N2—C7—C7^ii^	2 (2)
N1—C2—C3—C4	−177.9 (12)	C6—N2—C7—C8	−179.7 (12)
C1—C2—C3—C4	6 (2)	N2—C7—C8—Br3	−101.0 (12)
N1^i^—C3—C4—Br2	103.1 (11)	C7^ii^—C7—C8—Br3	77.4 (18)

Symmetry codes: (i) −*y*+1, −*x*+1, −*z*+1/2; (ii) *y*, *x*, −*z*.

### Hydrogen-bond geometry (Å, º)

**Table:**

*D*—H···*A*	*D*—H	H···*A*	*D*···*A*	*D*—H···*A*
C1—H1*A*···Br2^iii^	0.97	3.02	3.863 (14)	146
C1—H1*B*···Br2	0.97	2.86	3.617 (16)	135
C5—H5*A*···Br4^ii^	0.97	3.04	3.748 (16)	131
C5—H5*B*···Br3^iv^	0.97	3.03	3.864 (14)	145
C8—H8*A*···Br3^ii^	0.97	2.96	3.654 (15)	130

Symmetry codes: (ii) *y*, *x*, −*z*; (iii) −*y*+3/2, *x*−1/2, *z*+1/4; (iv) −*x*+1/2, *y*+1/2, −*z*+1/4.
